# Repeated intracoronary lithotripsy as a treatment for rebel stent underexpansion

**DOI:** 10.1002/hsr2.333

**Published:** 2021-08-03

**Authors:** José Valencia, Marta Herrero‐Brocal, Javier Pineda, Juan Miguel Ruiz‐Nodar

**Affiliations:** ^1^ Servicio de Cardiología Hospital General Universitario de Alicante Alicante Spain; ^2^ Unidad de Hemodinámica y Cardiología Intervencionista Hospital General Universitario de Alicante Alicante Spain

## 1 INTRODUCTION

Stent underexpansion during coronary angioplasty is a rare but difficult‐to‐manage complication that has been associated with an increase in stent thrombosis rates.^1^ Coronary intravascular lithotripsy (IVL) has represented a great advance in the treatment of calcified lesions; however, the results in underexpanded stents specific scenario are not satisfactory many times.

## 2 REPORT

**FIGURE 1:**
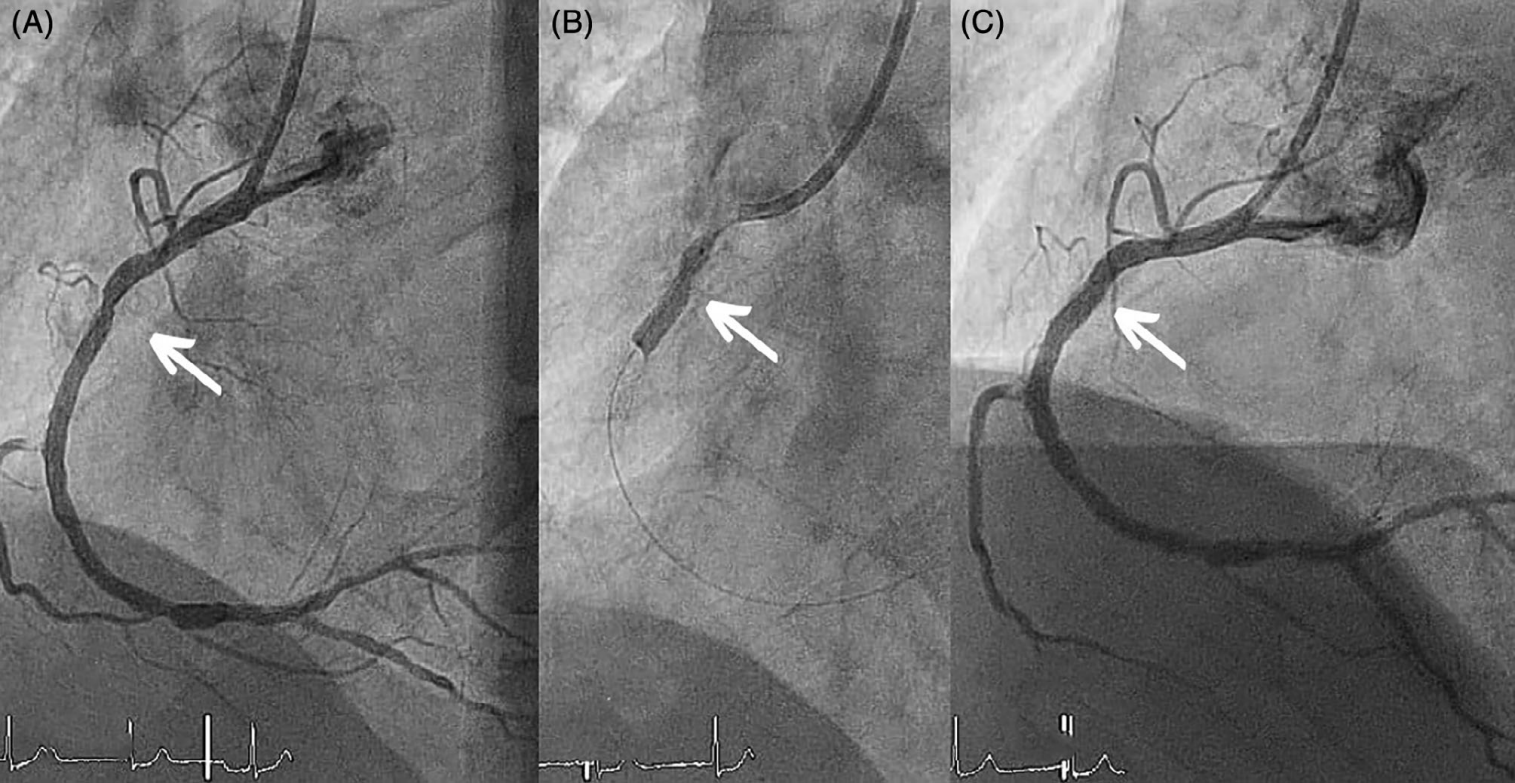
Right coronary artery angiography (RCA). (A) Primary angioplasty on the right coronary artery with a 3 × 32 mm stent implantation. The arrows indicate an underexpansion zone. (B) Therapy with a 3‐mm lithotripsy balloon. ShockwaveC2 balloon inflated to 4 atmospheres during the administration of shocks with a notch in the upper side indicating a zone of underexpansion. (C) Final angiographic result of the right coronary artery after repeated lithotripsy

A 62‐year‐old man consulted in the emergency room for chest pain. Electrocardiogram showed inferior ST elevation, so urgent coronary angiography was performed. Acute thrombotic occlusion of the middle right coronary artery was observed, therefore primary angioplasty was performed implanting a 3 × 32mm Cr‐Pt drug‐eluting stent at high pressure (20 atmospheres). An area of stent underexpansion was observed (Figure 1A), which persisted despite post‐dilation with high‐pressure balloons. Given the persistence of underexpansion, it was decided to perform IVL therapy with a 3 × 12 mm ShockwaveC2 balloon catheter with a theoretical 1:1 ratio to the vessel size (Shockwave Medical Inc. Santa Clara, CA), applying up to 80 shocks (all therapy allowed by the manufacturer), inflated to 4 atmospheres during therapy and rising to 6 atmospheres after each cycle, without achieving, despite this, a complete expansion (Figure 1B). After the procedure, the patient was admitted to the coronary unit already asymptomatic and with ST normalization.

**FIGURE 2:**
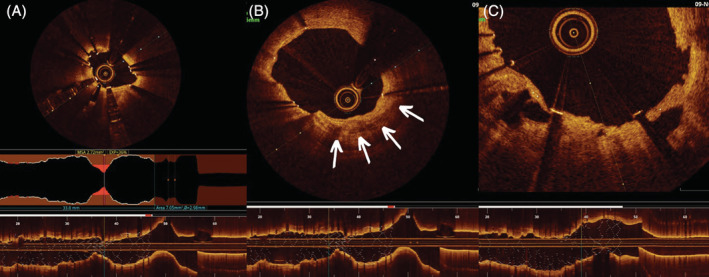
Optical coherence tomography study. (A) Zone of maximum underexpansion (36%) with luminal area of 2.72 mm^2^. (B) Extensive area of calcification visible from 2 to 7 (white arrows) in the distal segment adjacent to the area of maximum underexpansion. (C) Optical coherence tomography image showing the fracture points of the calcified plaque (white arrows)

After 72 hours, a second procedure was performed evaluating the underexpanded area with optical coherence tomography. A focal underexpansion of 64% was confirmed, with a minimum luminal area of 2.72 mm^2^ (Figure 2A). Extensive areas of calcification at the lesion level was confirmed (Figure 2B) as a cause of stent underexpansion. Given the risk of future stent thrombosis, it was decided to repeat IVL therapy with the ShockwaveC2 system, this time with a 3.50 × 12 mm balloon, applying again the maximum dose allowed of 80 shocks, inflated to 5 atmospheres during therapy and rising to 7 atmospheres after each cycle. During one of the last cycles, the plaque was fractured achieving complete expansion of the balloon (Figure 2C). The procedure was concluded with postdilation using a 3.75 mm noncompliant balloon with a good final angiographic result (Figure 1C).

## 3 DISCUSSION

Stent underexpansion represents a therapeutic challenge for the interventional cardiologist nowadays. The performance of intracoronary imaging studies to characterize the plaque and its adequate preparation with high‐pressure, cutting or scoring angioplasty balloons, prior to the implantation of the stent, reduces its incidence significantly. Despite this, the presence of calcification that is not appreciably visible on angiographies can result in the occurrence of said complication. Although IVL has shown very good results in the treatment of calcified de novo lesions, according to recent studies, in the specific scenario of stent underexpansion the results have been much worse, with success rates between 64.7%^2^ and 25%^3^ in different published series, and some isolated cases of stent thrombosis due to underexpansion treated with this therapy.^4^

The case we present here may present a possible solution to persistent stent underexpansion after IVL.

## 4 CONCLUSION

Repeated IVL therapy may be helpful in cases of rebel stent underexpansion that do not respond to a first cycle of treatment. More studies are needed to confirm these results.

## ETHICS STATEMENT

A signed informed consent was obtained from the patient and attached to this manuscript.

## CONFLICT OF INTEREST

The authors declare no conflicts of interest.

## AUTHOR CONTRIBUTIONS

Writing ‐ original draft preparation: José Valencia, Marta Herrero.

Writing ‐ review and editing: Juan Miguel Ruiz‐Nodar, Javier Pineda.

All authors have read and approved the final version of the manuscript.

José Valencia had full access to all of the data in this study and takes complete responsibility for the integrity of the data and the accuracy of the data analysis.

## TRANSPARENCY STATEMENT

The lead author (José Valencia), affirms that this manuscript is an honest, accurate, and transparent account of the study being reported that no important aspects of the study have been omitted; and that any discrepancies from the study as planned (and, if relevant, registered) have been explained.

## Data Availability

Data sharing is not applicable to this article as no new data were created or analyzed in this clinical case report.
